# Surface Microstructure of Two Bioceramics: Calcium-Enriched Mixture and Cold Ceramic in Setting Environments with Different pH Values

**DOI:** 10.1155/2023/7130619

**Published:** 2023-03-06

**Authors:** Maryam Kazemipoor, Ehsan Sanati

**Affiliations:** Department of Endodontics, School of Dentistry, Shahid Sadoughi University of Medical Sciences, Yazd, Iran

## Abstract

**Introduction:**

The pH of the setting environment could alter the surface characteristics of bioceramics. The present study aimed to assess the surface microstructure of calcium-enriched mixture (CEM cement) and cold ceramic (CC) in setting environments with different pH values.

**Materials and Methods:**

12 dentin blocks with 3 mm height and internal diameter were prepared. CEM cement and CC were prepared and packed into the blocks. Samples in each bioceramics group (*n* = 6) were divided into 3 subgroups (*n* = 2) and exposed to acid, pH of 7.4, and alkaline pH for 1 week. Specimens were prepared for evaluation under a scanning electron microscope using backscattered electron (BSE) detectors. Monitoring of pH changes was rendered with a pH meter through the setting process.

**Results:**

BSE detection in an acidic environment showed more amorphous microstructures in CC specimens in comparison to CEM cement. In pH of 7.4 and alkaline pH, more unhydrated structures were observed in CEM cement compared with CC samples. During the first 48 h of the setting process, the pH changes of setting environments were more rapid in the CEM cement group in comparison to CC samples.

**Conclusion:**

pH changes during the setting process of cement could affect the surface microstructure and physical properties. In acidic environments, the crystallization of CC cement is more disrupted than that of CEM cement.

## 1. Introduction

Bioceramic materials are a type of biocompatible ceramic that is highly suitable to be used inside the human body [1]. These materials have been used in root canal treatment since the 1990s [2]. Some properties of bioceramics materials include nontoxicity, lack of sensitivity towards moisture and blood, acceptable dimensional stability, minor expansion while setting, a perfect and long-term seal, biocompatibility, and an antibacterial effect [3]. These materials are capable of forming hydroxyapatite crystals and finally developing a bond between dentin and tooth structure [4]. Some applications of these materials in root canal treatment include sealing perforations, periapical surgery, pulp capping, pulpotomy, dental restoration of immature teeth with an open apex, teeth with internal/external resorption, and endodontic filling in regenerative endodontic therapy [5]. The wide spectrum of bioceramic materials includes Mineral Trioxide Aggregate (MTA), cold ceramic, CEM cement, biodentine, Portland cement, and Endosequence Root Repair Material (ERRM) [2, 6].

Mineral Trioxide Aggregate (MTA) is a type of bioceramic with various clinical applications, which is widely used in root canal treatment [5]. This bioceramic material is composed of dicalcium silicate, tricalcium silicate, tricalcium aluminate, gypsum, tetracalcium aluminoferrite, and bismuth oxide [3]. MTA advantages include high clinical success, a high potential for tissue regeneration, suitable sealing, and a good degree of biocompatibility [7]. On the contrary, the disadvantages of this material include its difficulty of use, long setting time, and the possibility of tooth discoloration as a result of using gray MTA [5]. Calcium-Enriched Mixture (CEM) cement consists of hydrophilic particles, and its constituent materials include calcium hydroxide, calcium carbonate, aluminum oxide, magnesium oxide, sulfur oxide, chlorine, water, and carbon dioxide [8]. PH values, operating times, and dimensional variations of CEM are quite similar to those of Pro Root MTA; however, lower film thickness, higher flow, and a lower setting time of CEM cement are among its superior physical properties compared to Pro Root MTA [8]. The antibacterial property of CEM cement is similar to that of calcium hydroxide, though it is relatively better than MTA and Portland cement [9]. Contamination with blood does not affect the sealing ability of CEM and MTA materials; however, CEM displays a higher sealing potential when contaminating human saliva [10].

Cold ceramic is a calcium hydroxide-based material. Its constituent materials are barium oxide, sulfur trioxide, calcium oxide, and silicon oxide. Cold ceramic displays a lower microleakage property compared with calcium hydroxide and amalgam [6]. Cold ceramics illustrated a better sealing ability in the environments as compared to MTA; however, their sealing potential is similar in both dry and salivary environments [11]. Just like calcium hydroxide and MTA, cold ceramic develops an alkaline pH. Its pH equals 7.36 after being mixed. This value reaches 10.11–10.84 after one or two hours and reaches 11.16 after a period of seven days [12].

To evaluate the properties of a bioceramic material, one must pay sufficient attention to its physical, chemical, and antimicrobial properties [13]. Some of its physical properties include setting time, hardness, compressive strength, and flexural strength [5]. Hardness is the material's resistance against surface deformities after receiving pressure and is one of the significant indicators in studying materials' general strength [14]. Factors such as the environment's pH, the material's thickness, condensation pressure, the amount of in-mixture trapped air, moisture, acidity, and temperature are among the factors contributing to the material's hardness [15].

Scanning Electron Microscopy (SEM) is considered a necessary tool for the description of nanometer- and micrometer-scaled materials. Also, it is one of the most varied instruments to study and analyze microstructure morphology, surface topography, crystallography, and available chemical compounds [16]. It has been illustrated that an acidic environment may affect both the physical and chemical properties of bioceramics, including their sealing ability, surface microhardness, and setting process [15]. In cases in which inflammation is present (e.g., abscess, *p*, pulp, and periapical inflammation), the pH level would be decreased [17]. The possible mechanism of this pH reduction *n* is the secretion of butyric acid, which is a byproduct of anaerobic bacteria's metabolism Because inflamed tissues are present in the environment at the time of application of a bioceramic material in root canal treatment, any change in pH value would lead to possible interferences in the setting process of bioceramic materials and their physicochemical properties [15]. The present study has been undertaken to evaluate and compare the microstructure of two bioceramics, CEM cement and cold ceramic, in setting environments with different pH values.

## 2. Materials and Methods

12 maxillary and mandibular single-rooted teeth, without any crack, fracture, or caries have been selected for this experimental study. Before the study begins, the teeth samples have been cleared of dental calculus, plaques, stain, and any soft or hard tissue connected to the dental surface through the use of a scaler (periodontal scaler TI-03-1015, Transact Int., Pakistan). A 2.5% sodium hypochlorite solution (Paksan, Iran) has been used for disinfecting dental samples for a period of 20 minutes. In the event of dental dehydration, the samples have been restored in a normal saline solution throughout the study at a temperature of 37°C.

A diamond saw microtome (SP1600 microtome; Leica, Nußloch, Germany) was used to obtain 12 root dentin slices with a height and internal diameter of 3 mm. No. 5 Peeso Reamer (Dentsply Maillefer, Ballaigues, Switzerland) (with a diameter of 1.5 mm) has been applied to prepare the internal space with a 3 mm diameter inside the canals. The resulting space was a cylinder with a diameter and height of 3 mm, respectively. These dimensions have been confirmed using a digital caliber (Mitutoyo Advanced Onset Sensor Digital Caliper, Kawasaki, Japan).

Two bioceramic materials, CEM cement (Yekta Zist Dandan, Iran) and cold ceramic (Yazd, Iran), have been prepared based on the manufacturer's instructions. The liquid has been gradually added to the powder using a spatula and has been mixed for 15–30 seconds. The working time for both mixtures has been defined as five minutes. After being mixed, both bioceramic materials have been inserted layer-by-layer in dentin blocks (12 samples) using an MTA carrier. Minimum condensation has been applied through manual pressure. Extra ceramic material has been removed from the surface of the sample using a wet cotton pellet.

The samples in each bioceramic material (6 samples) have been randomly assigned into three groups. The buffered butyric acid-soaked gas (pH = 4.4) and distilled water-soaked gas (pH = 7.4) have been used to produce acidic and neutral environments, respectively. Besides, potassium hydroxide-soaked gas (pH = 10.4) has been used to develop an alkaline environment. The samples have been completely wrapped in the aforementioned gas and stored for a period of one week at a temperature of 37°C [14, 16]. To monitor the level and variation of pH values, the three setting environments have been checked every 24 hours using a pH meter. To evaluate the effect of dentin buffering capacity at the different pH values, monitoring of pH was also rendered in dentin blocks without bioceramics and two bioceramics without dentin at the three values.

For microstructural analysis, the surface of the sample has been sputtered with a thin layer (20 nm) of gold to turn its surface from electrically unconducive to electrically conductive. Using the organic solution, any excess material has been cleared from the samples. The samples have been fixed using special glue, and their samples' surfaces have been scanned using SEM (PHENOM Pro X, Netherlands), in backscattered electron detector mode and surface topography, at a magnification ratio of 1000x and 4000x.

## 3. Results

Back Scattered Electron Detector (BSD) analyzes the relative density of various phases of two bioceramics that are set in a different environment as well as the presence/absence of hydrated/unhydrated grains.

**Figure 1:**
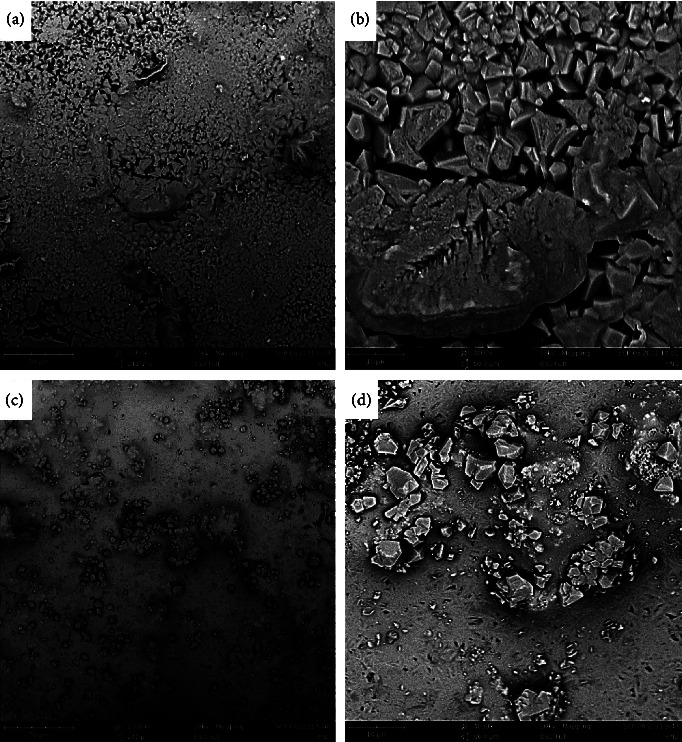
Scanning electron micrographs of CEM cement specimens after exposure to pH 4.4 (a), (b) at 2 different magnifications (×1000 and ×4000) using a backscattered electron detector. Scanning electron micrographs of cold ceramic specimens after exposure to pH 4.4 (c), (d) at 2 different magnifications (×1000 and ×4000) using a backscattered electron detector.

The BSE detector with a magnification ratio of 1000x in a sample of CEM cement stored in an acidic solution revealed big crystalline structures distributed on the surface of the material. Many pores have also been observed in the form of black areas. The light gray zones, which point to the presence of calcium hydroxide, are widely present, and a few dark gray zones have been observed, which detect the presence of the hydrated grains (Figure 1(a)).

In the SEM image of the cold ceramic stored in an acidic solution and with a magnification ratio of 1000, scattered crystalline structures have been observed within an amorphous texture. Besides, the surface was filled with bright grains, which detect the unhydrated zones (Figure 1(c)).

In the CEM cement sample stored in an acidic environment and with a magnification ratio of 4000x, the crystals are more evident and the number of amorphous zones is fewer compared to the cold ceramic material (Figure 1(b)).

The cold ceramic sample stored in an acidic environment and with a magnification ratio of 4000x illustrated a few crystallites within the wider amorphous context. In this magnification, the crystals were more prominent. The bright zones confirm the existence of unhydrated particles. The porosity among the developed crystals was well evident from this magnification (Figure 1(d)).

**Figure 2:**
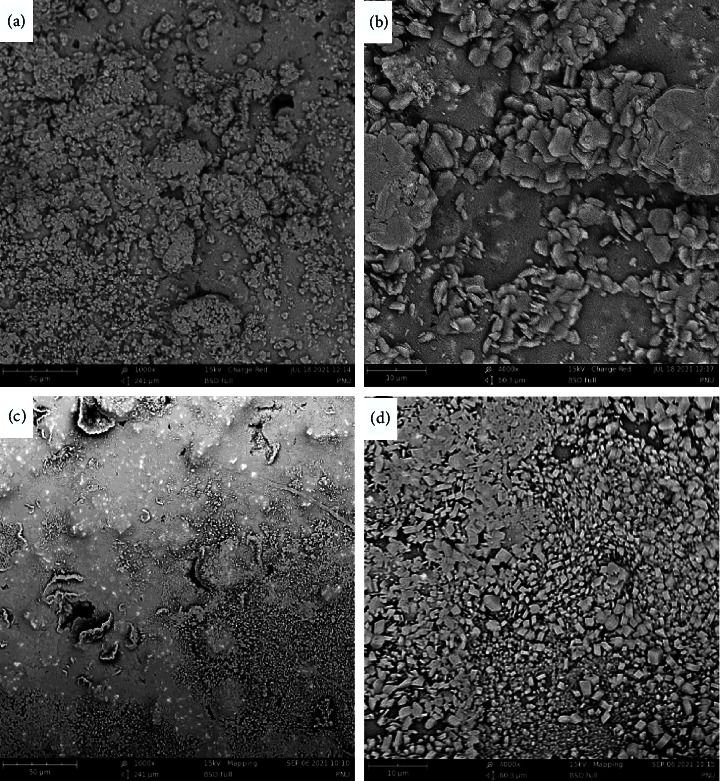
Scanning electron micrographs of CEM cement specimens after exposure to pH 7.4 (a), (b) at 2 different magnifications (×1000 and ×4000) using a backscattered electron detector. Scanning electron micrographs of cold ceramic specimens after exposure to pH 7.4 (c), (d) at 2 different magnifications (×1000 and ×4000) using a backscattered electron detector.

The SEM image of the CEM cement stored in a neutral environment with a magnification ratio of 1000x displays numerous crystalline structures next to the few amorphous structures. It is noteworthy that the crystal is not clear at this magnification. Moreover, few porosities were evident in this scan (Figure 2(a)).

The SEM image of the cold ceramic stored in a neutral environment with a magnification ratio of 1000x displayed crystalline structures next to the amorphous ones. Few porosities were evident in the amorphous context as well as scattered bright zones (Figure 2(c)).

The SEM image of the CEM cement stored in a neutral environment with a magnification ratio of 4000x displays numerous crystalline structures that are surrounded by amorphous ones. The crystals are well evident at this magnification. A few intercrystal porosities have also been observed (Figure 2(b)).

The SEM image of the cold ceramic stored in a neutral environment with a magnification ratio of 4000x displays numerous dense crystalline structures, which are distributed evenly over the surface in some regions. Amorphous structures and bright zones were also observed, but their numbers were limited. Low intercrystal porosity has also been observed. Dark gray grains, which display the presence of other hydrated materials, have been observed in this image as well (Figure 2(d)).

**Figure 3:**
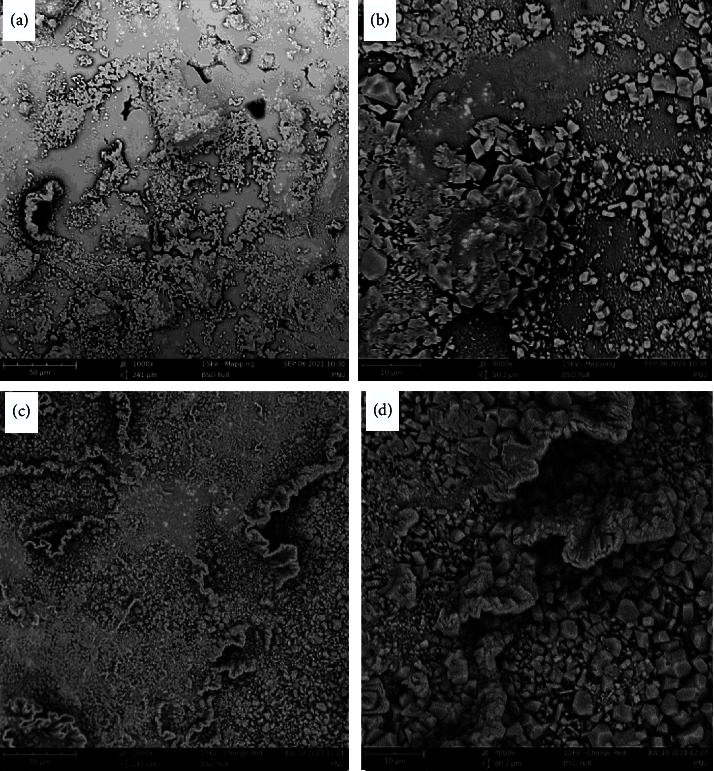
Scanning electron micrographs of CEM cement specimens after exposure to pH 10.4 (a), (b) at 2 different magnifications (×1000 and ×4000) using a backscattered electron detector. Scanning electron micrographs of cold ceramic specimens after exposing them to pH 10.4 (c), (d) at 2 different magnifications (×1000 and ×4000) using a backscattered electron detector.

The SEM image of the CEM cement stored in an alkaline environment with a magnification ratio of 1000x displayed numerous crystalline structures within the amorphous context. In addition, porosity was present between crystals as well as within the amorphous context (Figure 3(a)).

The SEM image of the cold ceramic stored in an alkaline environment with a magnification ratio of 1000x displayed dense and wide crystalline structures without the amorphous structure; however, the crystals were not clear in this image (Figure 3(c)).

The SEM image of the CEM cement stored in an alkaline environment with a magnification ratio of 4000x displayed dense crystalline structures within an amorphous context. Low porosity has been observed between crystals. Besides, unhydrated bright zones have also been observed in amorphous structures (Figure 3(b)).

The SEM image of the cold ceramic stored in an alkaline environment with a magnification ratio of 4000x displayed numerous dense crystalline structures, which are completely evident in some regions. The amorphous structure was limitedly observed. Low intercrystal porosity has been observed in this image (Figure 3(d)).

**Figure 4:**
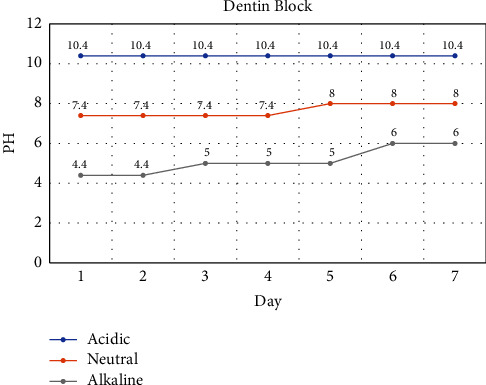
Monitoring of pH values in dentin block reserved in different setting environments.

In monitoring pH variations within a week, no variations have been observed in the dentine block stored in an alkaline environment with no bioceramic material present. The pH variations in the neutral environment were 7.4 to 8 in a one-week interval. Moreover, the pH variations in the acidic environment were 4.4 to 6 within a one-week interval (Figure 4).

**Figure 5:**
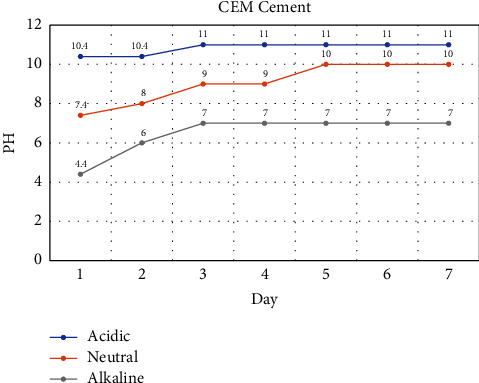
Monitoring of pH values in CEM cement samples reserved in different setting environments.

In the CEM cement bioceramics group, pH variations during the first 3 days of the setting process in alkaline, neutral, and acidic environments were 10.4 to 11, 7.4 to 9, and 4.4 to 7 with an ascending trend from the first day, respectively (Figure 5).

**Figure 6:**
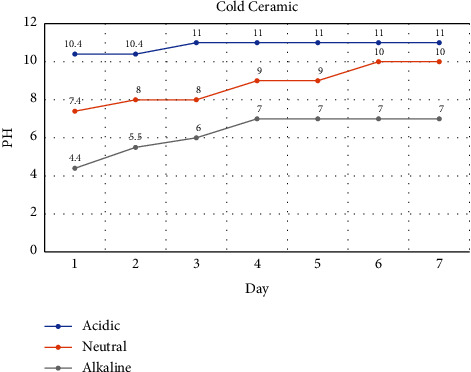
Monitoring of pH values in cold ceramic samples reserved in different setting environments.

In the cold ceramic bioceramics group, pH variations during the first 3 days of the setting process in alkaline, neutral, and acidic environments were 10.4 to 11, 7.4 to 9, and 4.4 to 7 with an ascending trend from the first day, respectively (Figure 6).

## 4. Discussion

The physical and chemical properties of bioceramics are influenced in environments with different pH values [18]. Early hydration, development of hydrate phases, and reactions of cement clinkers may be influenced by the concentration of hydrogen ions and pH [19]. Important mechanical and chemical properties of a biomaterial, i.e., setting reaction, compressive and tensile strength, hardness, and microleakage, may be impaired in low or high pH environments [3, 14, 20, 21]. The effect of pH on the properties of fully set bioceramics has been studied using two methods: the pH of the setting environment and using high-pH solutions as a liquid phase for the acceleration of the setting [22, 23].

In the clinical conditions in which bioceramics are applied, the presence of inflammation usually creates an acidic environment. In the presence of pulpal and periradicular inflammation, the normal pH value of 7.4 tends to become acidic [17]. Tronstad et al. [24] revealed that in normal pulp, the pH value of pulp, dentine, cementum, and periradicular tissue is in the range of 6.4 to 7.

On the other hand, Martínez-Ramírez and Palomo [25] reported that a high concentration of NaOH during the hydration phase of Portland cement may lead to the breakdown of calcium silicate gel. High alkalinity in the clinical condition could occur after the placement and removal of an intracanal medicament like calcium hydroxide. After the placement of calcium hydroxide during root canal therapy, the pH value of root dentine changed from 11.1 to 12.2 [24].

During the application of bioceramics in clinical conditions, pH changes due to the interaction of the setting environment and bioceramics are unavoidable. Various bioceramics, due to the ingredients and mechanism of setting, may behave differently against pH changes. It seems that during the early phase of hydration, the setting process and crystallization of bioceramics are more affected [26]. Moreover, monitoring the pH changes during the setting process could help the researchers explain the different crystallization behaviors of bioceramics. Since there are no data on the crystallization behavior of other currently available bioceramics at different pH values, the present research aimed to assess the surface microstructure of two bioceramics: a calcium-enriched mixture and a cold ceramic in setting environments with different pH values.

In the present study, to simulate the clinical condition, the samples were immersed in pieces of gauze soaked with either neutral, acidic, or alkaline solutions. All the material placements were done with a hand condenser and pressure to avoid gaps and voids in the material [27].

According to the pH changes during the first 48 h of the setting process, the most rapid increase in pH value was observed in CEM cement samples immersed in acid (4.4 to 7), followed by neutral (7.4 to 9) and alkaline (10.4 to 11) conditions. In contrast, CC samples showed a lower increase in pH value in an acidic (4.4 to 6) or neutral (7.4 to 8) environment.

It seems that the duration to reach a neutral pH value is longer in CC (4 days) samples in comparison to CEM cement (3 days). Within the first 48 h of the setting reaction, the pH value of the acidic and neutral environment is higher in CEM cement than in the CC group.

Backscattered electron (BSE) scanning is defined as high-energy electrons that are backscattered from a substrate's depth. The BSE modality in scanning electron microscopes is applied to analyze the relative densities of different phases of a microstructure based on the atomic number of elements in the cement [28]. Since in the present study, evaluation of the presence and proportion of different phases was important in different setting environments we have used this modality with two different magnifications for better assessment of the changes.

Previous studies have shown that in BSE images obtained from white MTA, four distinguishable phases were present [28, 29]. Bright grains, light gray, darker gray, and black areas represent grains of unhydrated cement, calcium hydroxide, other hydration products, and pores, respectively [28–30]. Calcium hydroxide is the main product after the hydration of bioceramics [31]. In the present study at high magnification, the hydrated grains, including light gray and gray structures, were denser and bigger with higher irregularity and pores in CEM that hardened in the acidic environment compared with the CC acid group. The CC acid group revealed higher unhydrated areas concerning CEM cement samples. CEM cement samples either set in neutral or alkaline environments showed smaller crystals distributed over the surface. Unhydrated areas were also observed between the crystals, and the density of the crystals was higher and more homogenous in the CC alkaline group. The highest density and distribution of hydrated areas and the lowest of unhydrated ones were observed in the CC neutral and alkaline groups.

Giuliani et al. [30] studied the effect of pH on surface hardness and microstructure of WMTA and aureole. Based on the results, WMTA had more irregular and bigger hydrated grains in an acid environment compared with the pH 7.4 group. The aureole group was more sensitive to an acid pH environment and at neutral pH, aureole showed more homogenous rounded grains in comparison to more crystalline formation in WMTA.

The size and irregularity of WMTA in the acid group were similar to the BSE records of the CEM cement group in the present study. It seems that, like aureole, CC is also more sensitive to an acid environment.

Saghiri et al. [23] assessed the SE micrographs of MTA in an alkaline setting environment. Results showed that WMTAs exposed to pH values of 7.4 and 10.4 had more porosity and unhydrated areas. At a pH value of 8.4, WMTA had been hydrated completely.

Calcium hydroxide is a widely used intracanal medicament for its antibacterial activity and hard tissue formation [32]. Studies have shown that calcium hydroxide medication could adversely affect bioceramics' sealing ability and mechanical properties [33, 34]. It has been documented that high concentrations of NaOH in the hydration solution of Portland cement may lead to gel breakdown and influence the physical properties of cement [35].

In the present study, for the first time, we have applied dentin blocks as a matrix for bioceramics placement and to mimic the clinical condition. The buffering capacity of dentin could affect the pH values of the setting environment. Based on the results of the present study, pH changes during the first three days of the setting process in dentinal blocks without bioceramics showed an increase (4.4 to 5) only in an acid environment. The presence of dentinal blocks in the present study along with the different compositions of two bioceramics might lead to different surface views and crystallization. As bioceramics are capable of forming hydroxyapatite crystals and finally developing a bond between dentin and tooth structure, it would be interesting to test these materials in combination with biomimetic hydroxyapatite. Additionally, recent research showed that biomimetic hydroxyapatite compounds showed deposition of hydroxyapatite on polymeric composites [36]. It would be interesting in the future to test bioceramics in combination with this emerging material for clinical applications.

We have applied butyric acid, a product of anaerobic bacteria, to simulate the inflammatory condition that occurred in the clinical situation and the presence of periradicular infection [14].

The presence of voids, air bubbles, and pores could interfere with the sealing ability of bioceramics, especially in the margins and interface of bioceramics and dentin [37]. In the present study, the CEM cement acid group showed the highest number of pores on the surface because of the size of the crystals.

Considering the clinical condition where a bioceramic is set, there are many neutralizing factors that play an important role in the changes in pH in the setting environment. Since we could not simulate the total clinical condition in an in vitro study, the analysis of the data was limited to the condition of the setting environment rather than actual clinical settings.

## 5. Conclusion

Within the limitations of the present study, besides the attempts to simulate the clinical condition, in the presence of acidic material, CEM cement showed better results in comparison to CC. It might be due to the first rapid increase in pH values after CEM cement placement. CC, as opposed to CEM cement, showed better results in neutral and alkaline setting environments. It seems that the rate of changes in pH values during the early phase of hydration could influence the setting process and crystallization of bioceramics. Future studies in this regard could help the researchers better analyse the behavior of bioceramics in different setting environments.

## Data Availability

All the fundamental data are included in the text. The data supporting the findings of this study will also be available from the corresponding author on request.
